# Synthesis of Polyaniline in Seawater

**DOI:** 10.3390/polym12020375

**Published:** 2020-02-07

**Authors:** Takuya Yonehara, Kyoka Komaba, Hiromasa Goto

**Affiliations:** Department of Materials Science, Faculty of Pure and Applied Sciences, University of Tsukuba, Tsukuba, Ibaraki 305-8573, Japan; s1820417@s.tsukuba.ac.jp (T.Y.); s1920388@s.tsukuba.ac.jp (K.K.)

**Keywords:** polyaniline, conducting polymer, seawater

## Abstract

To date, polyaniline (PANI) has been synthesized in pure water. Aside from this, the application of PANI as a conducting polymer could be extended if it can be effectively synthesized in seawater, which constitutes 70% of the surface of the Earth. The production of functional plastics using natural resources without any additional purification would improve industrial production and enhance the comfort associated with our daily life. However, no examples of the effective application of seawater to PANI synthesis have been reported. Herein, PANI with an electrical conductivity of ~10^−2^ S/cm was synthesized in seawater as the reaction solvent. The electron spin resonance measurements confirmed the role of the polarons of PANI as charge carriers. In addition, a PANI/silk composite was prepared in seawater to produce a conducting cloth for further applications. The performance of the PANI prepared in seawater as the solvent was comparable to that of the PANI prepared in pure water. Thus, the proposed method allowed for the production of the conducting polymer via a convenient and low-cost method. This is the first study to report the usage of seawater as an abundant natural resource for synthesizing conducting polymers, promoting their wide application.

## 1. Introduction

Conducting polymers have attracted considerable attention because of their unique magnetic and electrical properties. They have been used in several electronic and energy devices, including solar cells, and the electrical conduction plastics [1,2,3,4]. However, the highly developed π-conjugation system of conducting polymers causes low processability. Even though this drawback can be compensated by introducing a flexible alkyl group, the resulting steric hindrance increases the dihedral angle between the monomer repeat units, which decreases the intra-and inter-chain interactions. Therefore, further optimization is required during molecular design to introduce an alkyl group into the main chain of a conjugated polymer and to increase the processability as well as improve the intrinsic electrical properties. Alternatively, non-substituted conducting polymer composites with good processability can be prepared [5].

The production of pure water is important for our daily lives, and the demand for pure water in industry has increased owing to the development and progress of the mass production of fine electronic devices, chemicals, and medicines. To satisfy the demand for pure water, the utilization of seawater, which is natural and abundant product, is proposed. However, few examples of the effective application of seawater for plastic technology applications have been reported. 

Polyaniline (PANI) is a unique conducting polymer. The electrical conductivity of PANI can be attributed to polarons, which serve as charge carriers and ionic conductors. Generally, PANI is synthesized in pure water without an inert gas atmosphere; however, the development of a more convenient synthesis process for PANI would be promising for industrial applications [6,7,8]. To date, PANI has been synthesized using pure water as the polymerization solvent, and no reports have investigated the preparation of PANI in seawater. The wide application of PANI as a conducting polymer would be possible if PANI could be synthesized in natural seawater. Although, the trace amount of metal ions in seawater is dependent on the region, the salt composition is observed to remain constant in the sea (i.e., the salt composition in seawater: NaCl: 77.9%, MgCl: 9.6%, MgSO_4_: 6.1%, CaSO_4_: 4.0%, and KCl: 2.1% [9]). This research reports the synthesis of PANI in natural seawater instead of distilled water. The application of seawater for the production of PANI allows a convenient and inexpensive synthetic process, which can be used for the mass production of conducting polymers. Furthermore, this simple methodology may connect polymer synthesis and ocean technology in the future.

## 2. Materials and Methods

**Figure 1:**
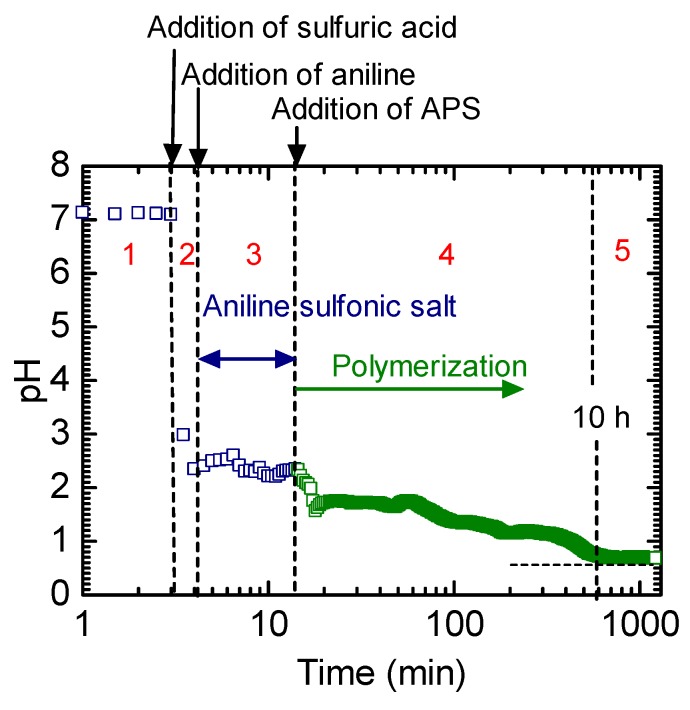
Change in pH during polymerization. Step 1: The seawater pH values before the reaction. Step 2: The change in pH after the addition of sulfuric acid. Step 3: The change in pH after the addition of ammonium peroxodisulfate (APS). Step 4: The change in pH after the addition of APS to the polymerization process to obtain polyaniline. Step 5: The completion of polymerization. The solution pH value was stabilized. The steps are designated with red colored numbers.

Aniline (1 g, Wako Ltd, Osaka, Japan), as the monomer, and sulfuric acid (1 mL, Nacalai Tesque Inc, Kyoto, Japan) were dissolved in seawater (300 mL, Shimoda Gulf, Japan). After cooling to 0–5 °C using an ice bath, ammonium peroxodisulfate (APS, 1 g, Yoneyama Kogyo Ltd, Osaka, Japan) was added to the mixture for initiating polymerization, and the change in pH was monitored. The pH gradually decreased during polymerization, and this decrease with time could be defined in five steps, as illustrated in Figure 1. The pH of seawater is considered to be neutral (approximately 7) (Step 1). The addition of sulfuric acid drastically decreased the pH (Step 2), and the subsequent addition of aniline to form aniline sulfuric acid further decreased the pH of the solution (Step 3). Furthermore, the addition of APS for polymerization resulted in a pH lower than 2 (Step 4). The polymerization of aniline in seawater was stabilized and completed after ~10 h (Step 5). The solution was subsequently filtered and dried under a reduced pressure to obtain a dark, emerald-green-colored PANI powder. PANI prepared in seawater and pure water (as a reference) was abbreviated as PANI_SEA_ and PANI_H2O_, respectively. The polymerization of aniline in seawater was conducted in the presence of silk cloth (JIS standard silk cloth) to prepare a PANI/silk composite with a thickness of 0.16 mm.

As previously mentioned, the salt concentration in seawater and its composition in metal ions are generally stable. Therefore, except for minor effects due to the metal ion traces, no regional dependency should be expected for the PANI synthesized in seawater. In addition, the seawater of Shimoda Gulf is a standard in constitutions because it is considered to be the temperate zone in the world.

## 3. Results

### 3.1. IR

**Figure 2:**
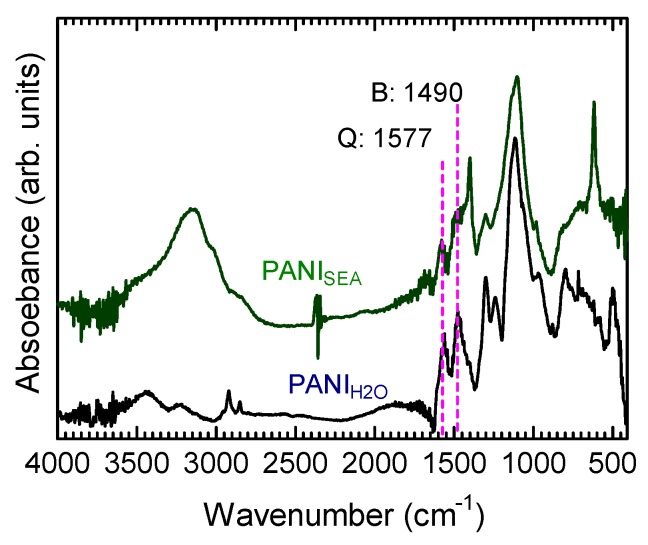
FT–IR spectra of the samples prepared using seawater as the solvent (polyaniline prepared in seawater, PANI_SEA_) and the standard PANI (polyaniline prepared in distilled water, PANI_H2O_) prepared using pure water as the solvent.

**Figure 3:**
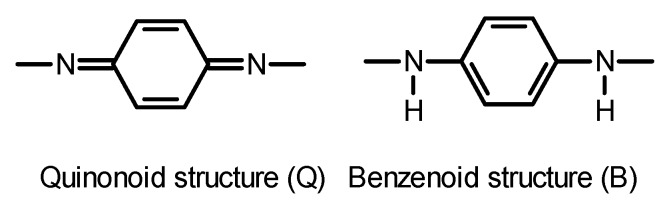
The quinonoid and benzenoid structures in the main chain of PANI.

The broad signal near 3000–3300 cm^−1^ originated from the N–H stretching vibration, whereas the signals at 1577 and 1490 cm^−1^ were attributed to the quinonoid and benzenoid structures, respectively (Figure 2 and Figure 3). The absorption bands of PANI_SEA_ were similar to those of PANI_H2O_, which was used as the standard, except for the absorption bands at 1406 and 616 cm^−1^, which may be attributed to the impurities in natural seawater. The signal around 1250 cm^−1^ originated from the C–N stretching vibration of the aromatic amine. Although it was currently difficult to compare the waveforms of the region 500 to 1000 cm^−1^, it can be related to the structures of the polymers containing trace amounts of seawater elements. These results indicated that PANI_SEA_ can be successfully synthesized in natural seawater. Although the resultant PANI_SEA_ at the *N* position of the polymer could partly coordinate with the trace amounts of seawater elements (Fe, Mn, Cr, Ni, Cu, Zn, Cd, and Pb), the effect of these elements did not apparently change the electromagnetic property of PANI_SEA_. Furthermore, the appearance of an absorption band at 1140 cm^−1^ can be attributed to the doped structure, indicating that the prepared PANI_SEA_ sample was a doped state of the PANI conducting polymer [10].

### 3.2. Electrical Conductivity and Electron Spin Resonance

**Figure 4:**
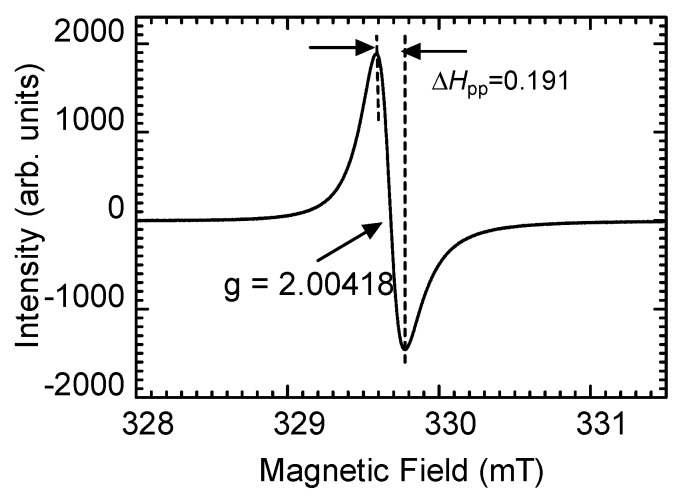
The electron spin resonance of the PANI_SEA_ prepared using seawater as the solvent.

The electrical conductivity of the pressed pellet form of PANI was ~10^−2^ S/cm, confirming that it is a conducting polymer. The electron spin resonance (ESR) spectroscopic recordings on the powdered PANI_SEA_ sample detected an unpaired electron as a polaron (charge carrier). Furthermore, microwave radiation from the ESR during the measurement was absorbed to the surface sample as a skin effect. A Dysonian-type asymmetric line shape was observed in the ESR spectrum, indicating the high electrical conductivity of the material. The peak-to-peak linewidth (Δ*H*_pp_) and the g values were 0.191 mT and 2.00418, respectively (Figure 4). Our previous study demonstrated that Ca^2+^/PANI_H2O_ exhibits electrical conductivity in the range of 10^−^^3^ S/cm with a high Ca^2+^ content. However, an increase in the Ca^2+^ component vs. the aniline monomer during the synthesis of PANI reduced the synthetic yield. Regardless, PANI_SEA_ exhibited higher conductivity when compared with that exhibited by Ca^2+^/PANI_H2O_, indicating that the performance of the resultant PANI_SEA_ was not significantly affected by the trace amounts of metal ions.

### 3.3. Scanning Electron Microscopy

**Figure 5:**
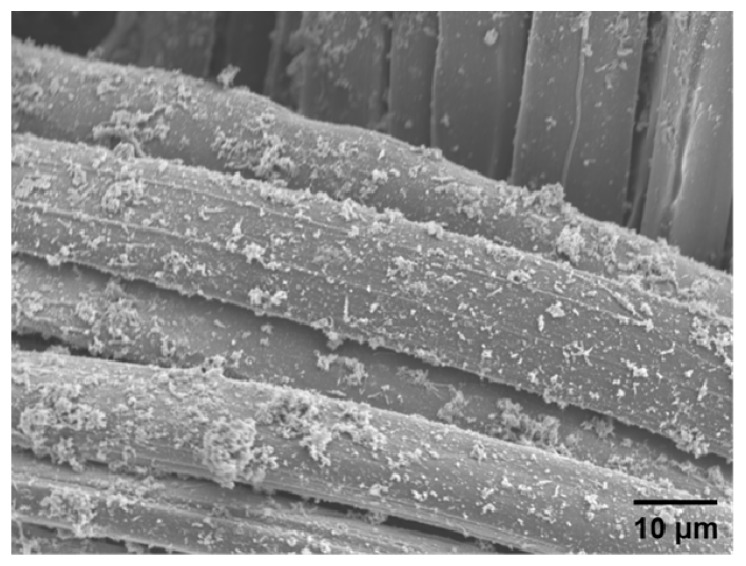
Scanning electron microscopy (SEM) image of the PANI_SEA_/silk composite prepared using seawater.

The surface of the PANI_SEA_/silk composite was investigated via scanning electron microscopy (SEM) (Figure 5). The silk textile surface was covered with PANI_SEA_ and cotton-like PANI_SEA_ aggregates deposited on the surface, indicating that the conducting polymer composite textile was successfully prepared in seawater with electrical conductivity of 10^−^^2^ S/cm as a moderate electrical conductivity.

## 4. Discussion

In this study, we demonstrated that the seawater components, such as organic materials and minerals, do not affect the polymerization activity of the aniline monomer during the formation of PANI_SEA_ as a conducting polymer. This implies that other types of conducting polymers, such as polypyrrole, polythiophene, and poly(3,4-ethylenedioxythiophene) (PEDOT), could also be conveniently prepared in seawater to ensure their large-scale production. The appearance of Dysonian-type signals that are generally observed for graphene in the ESR spectra indicated that the resultant polymer is a good electrical conducting material. Furthermore, the narrow peak-to-peak width in the ESR implied the delocalization of the charge carriers along the polymer main chain.

## 5. Conclusions

PANI was synthesized in a natural seawater solution without demineralization via oxidative polymerization. The electrical conductivity of PANI_SEA_ was 10^−2^ S/cm, which is a moderate value when compared to that of PANI_H2O_, indicating that aniline can be polymerized in seawater in the presence of biological materials and metal ions. This result can be of considerable significance for the polymer industry and the generation of conducting polymers because PANI can be produced using low-cost and convenient technique. In addition, the polymerization of aniline in the presence of silk via this simple method afforded a PANI/silk composite, which is a conducting cloth material that can be used as an electromagnetic shield, an anti-corrosion sheet, and an anti-electrostatic wiper.
